# Prenatal Diagnosis of Isolated Single Umbilical Artery: Incidence, Risk Factors and Impact on Pregnancy Outcomes

**DOI:** 10.3390/medicina59061080

**Published:** 2023-06-03

**Authors:** Antonios Siargkas, Sonia Giouleka, Ioannis Tsakiridis, Apostolos Mamopoulos, Ioannis Kalogiannidis, Apostolos Athanasiadis, Themistoklis Dagklis

**Affiliations:** Third Department of Obstetrics and Gynecology, School of Medicine, Faculty of Health Sciences, Aristotle University of Thessaloniki, 541 24 Thessaloniki, Greece; antonis.siargkas@gmail.com (A.S.); soniagiouleka@outlook.com (S.G.); amamop@auth.gr (A.M.); ikalogiannidis@gmail.com (I.K.); apostolos3435@gmail.com (A.A.); tdagklis@gmail.com (T.D.)

**Keywords:** single umbilical artery, small-for-gestational-age, preterm delivery, risk factors, pregnancy outcomes

## Abstract

*Objectives*: To assess the incidence of prenatally diagnosed isolated single umbilical artery (iSUA) and its impact on major pregnancy outcomes, as well as to investigate potential risk factors. *Materials and methods*: A prospective study of singleton pregnancies, undergoing routine anomaly scans at 20^+0^–24^+0^ weeks of gestation, was carried out from 2018 to 2022. The effect of sonographically detected iSUA on small-for-gestational-age neonates (SGA) and preterm delivery (PTD) was evaluated using parameterized Student’s *t*-test, nonparametric Mann–Whitney U test and the chi-square test. Multivariable logistic regression models were implemented to assess the independent association between iSUA and the main outcomes, as well as with potential risk factors, while adjusting for specific confounders. *Results*: The study population included 6528 singleton pregnancies and the incidence of prenatally diagnosed iSUA was 1.3%. Prenatally diagnosed iSUA had a statistically significant association with both SGA neonates (aOR: 1.909; 95% CI: 1.152–3.163) and PTD (aOR: 1.903; 95% CI: 1.035–3.498), while no association was identified between this sonographic finding and preeclampsia. With regard to risk factors, conception via assisted reproductive technology (ART) was associated with increased risk of iSUA (aOR: 2.234; 95% CI: 1.104–4.523), while no other independent predictor for the development of this anatomical variation was identified. *Conclusions*: Prenatally diagnosed iSUA seems to be associated with a higher incidence of SGA and PTD and is more common in pregnancies following ART, which constitutes a novel finding.

## 1. Introduction

Single umbilical artery (SUA) is a variation of the umbilical cord anatomy where there is only one artery around the fetal bladder and in the fetal umbilical cord [1], whereas typically, the umbilical cord contains two arteries and one vein surrounded by the Wharton’s jelly and covered by a single layer of amnion [2]. The incidence of SUA is reported to be between 0.4% to 1%, depending on the population studied [3]; it is caused by either secondary atresia or atrophy of a previously normal umbilical artery, primary agenesis of one of the umbilical arteries or persistence of the original single allantoic artery of the body stalk [1]. 

While an apparently isolated SUA (iSUA) is not strongly correlated with chromosomal abnormalities, the coexistence of one or more anatomical fetal defects increases the risk of aneuploidy, especially trisomy 18 [4]. Notably, congenital malformations associated with SUA include mainly cardiac and renal defects [5]. According to recently published data, prenatally diagnosed iSUA carries a higher risk of fetal growth restriction, stillbirth, preterm delivery and small-for-gestational-age neonates (SGA) [6,7]. Nonetheless, there is currently no strong recommendation on routine screening for SUA or specific management, in case of diagnosis [8]. Additionally, there is limited research regarding the risk factors of iSUA; to date, white race, female sex of the neonate and maternal primigravidity have been reported in the literature as risk factors, whereas African-American race seems to have a protective role [9,10,11].

Therefore, the aim of this prospective study was to investigate the impact of diagnosing iSUA during the prenatal period; we aimed to assess the risk factors and possible adverse pregnancy outcomes of these cases in a well-designed setting and offer exact and trustworthy effect estimates for potential associations.

## 2. Materials and Methods

### 2.1. Study Design, Participants

A prospective study was carried out from June 2018 to June 2022, at the Third Department of Obstetrics and Gynecology, School of Medicine, Aristotle University of Thessaloniki, Greece. The study’s design and reporting adhered to the STROBE checklist [12].

All women experiencing a singleton pregnancy and undergoing routine anomaly scan at 20^+0^–24^+0^ gestational weeks were eligible to participate in the study. Pregnancies with aneuploidy or congenital anomalies, as well as with miscarriage or termination of pregnancy and those with missing data were excluded from the analysis. Maternal–fetal medicine specialists of the Department performed all the scans. Pregnancies diagnosed with iSUA were compared to those with a three-vessel cord, serving as study and control groups, respectively. The diagnosis of SUA was performed by direct visualization of the umbilical cord, or by tracking the umbilical arteries around the fetal bladder with color Doppler, as recommended by the International Society of Ultrasound in Obstetrics and Gynecology [8]; all the cases of SUA were postnatally confirmed by direct visualization of the cord. The fetal crown–rump length measurement in the first-trimester scan was used for accurate pregnancy dating in spontaneous conception and date of embryo-transfer in cases following assisted reproductive technology (ART). The patient data, including demographics and obstetric characteristics, were stored in a specialized database (Astraia Software GmbH, Munich, Germany).

All the participants authorized the use of their anonymized files for the study via a written consent form. The bioethics committee of the School of Medicine at the Aristotle University of Thessaloniki, Greece, approved the study protocol (3.188/2-5-2018). It should be noted that there were no benefits offered for participating in the study.

### 2.2. Measurements

In the context of this study, medical and obstetric parameters were collected. All the relevant covariables used in our analysis, including sociodemographic characteristics (age, parity, smoking, method of conception and body mass index-BMI), were collected during the recording of the maternal history or directly through our database.

Our study’s analyses focused on SGA, defined as a birthweight below 10 st., and preterm delivery (PTD), defined as birth between 22 and 37 weeks of gestation. We also investigated the impact of iSUA on preeclampsia, as well as the possible risk factors that could be correlated with the presence of iSUA.

During the routine anomaly scan, uterine artery pulsatility index z-score (UtA PI z-score) was recorded as per protocol [8], as well as any other umbilico-placental variation, such as abnormal cord insertion (marginal or velamentous).

### 2.3. Statistical Analysis

Comparisons between groups were carried out using the parameterized Student’s *t*-test and nonparametric Mann–Whitney U test for continuous variables and the chi-square test for the categorical ones. The continuous variables are described as means and standard deviation when normally distributed, and otherwise as medians and interquartile ranges. Population and percentages are used to represent categorical variables.

Taking into consideration the known and available confounders, associations with the two primary outcomes, SGA and PTD, were investigated using both univariate and multivariable logistic regression models [13,14]. Of note, the most recent suggestions state that in order to determine the unbiased prognostic influence of the new factor, a regression model that incorporates all of the previously recognized factors is required [15]. To date, variables such as maternal age, BMI, smoking, multiparity, conception via ART and history of miscarriage or preterm birth are well-established factors of PTD [14,16]. In addition to these, variables that are suspected to affect PTD rates, such as bleeding during the first trimester, abnormal cord insertion and abnormal UtA z-score were also included in our model [17,18,19,20]. Since most of these factors have also been linked to SGA, we decided to include them all in the corresponding models, thus making it simpler to compare our findings [13]. Multicollinearity was assessed by calculating the variance inflation factor for the variables of every model; we also investigated the existence of any influential observations that could distort our results.

To account for confounding and provide more accurate effect estimates, a risk factor analysis using a multivariable logistic regression with the iSUA as the dependent variable was carried out. The backward stepwise selection method was used to identify the risk factors with a significant prognostic value for iSUA.

The TRIPOD recommendations were applied while conducting and reporting the statistical analysis [21]. All analyses were performed in R 2.15.1 (R foundation, Vienna, Austria).

## 3. Results

**Figure 1:**
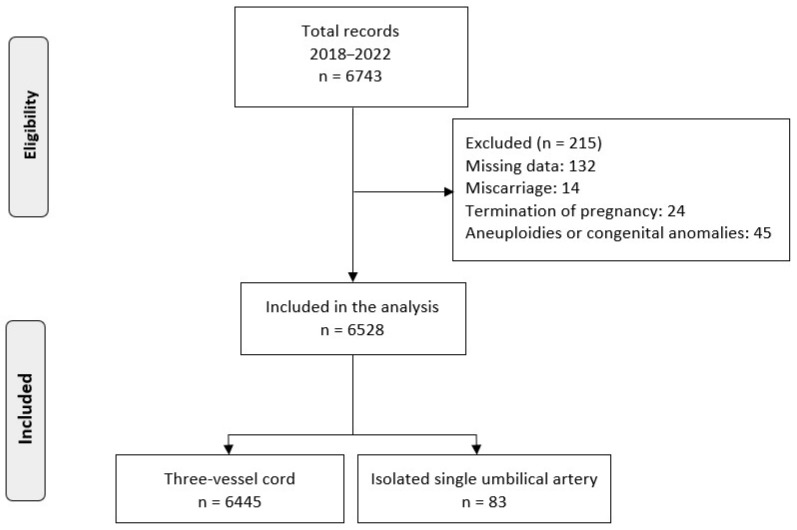
Study population selection.

The study sample consisted of a population of 6528 singleton pregnancies, after excluding 215 due to missing data (*n* = 132), miscarriage (*n* = 14), pregnancy termination (*n* = 24) and aneuploidies or congenital anomalies (*n* = 45) (Figure 1). Prenatally diagnosed cases of iSUA were detected in 1.3% (83/6528) of all pregnancies. It was observed that several of the investigated factors differ between the two groups. In particular, compared to the control group, the study group had a higher incidence of conception via ART (10.8% vs. 4.9%; *p* = 0.025) and higher rates of SGA (27.7% vs. 16.2%; *p* = 0.008) and PTD (16.9% vs. 8.4%; *p* = 0.011). Moreover, iSUA was associated with lower median values for gestational age (38.3 vs. 39.0 gestational weeks; *p* < 0.001) and lower median birthweight (3000 vs. 3250 g; *p* < 0.001). Regarding preeclampsia, 6 women out of 83 in the study group (7.2%) and 480 women out of 6445 in the control group (7.4%) were diagnosed with it; no association between iSUA and preeclampsia was detected (*p* = 1.0) (Table 1).

The univariate logistic regression model found that iSUA was associated with twice the risk for SGA neonates compared to singleton pregnancies with a three-vessel cord (OR: 1.958; 95% CI: 1.222–3.225). The association persisted in the multivariable model, pointing to a 1.9 times higher probability for SGA (aOR: 1.909, 95% CI: 1.152–3.163). Increased BMI and multiparity were important confounders, reducing the risk of SGA, while current smoking and an increased UtA PI-z score had a positive association with SGA. A higher probability for PTD was also identified in the study group compared to the controls, both in the univariate (OR: 2.201; 95% CI: 1.231–3.936) and the multivariable analysis (aOR: 1.903; 95% CI: 1.035–3.498). Regarding PTD, six variables were identified as significant confounders; increased BMI, current smoking, ART, increased UtA PI z-score, previous history of 1st trimester miscarriage and previous history of PTD, all having a positive association with the risk of PTD (Table 2, Supplementary Tables S1 and S2).

Regarding risk factor analysis, conception via ART was found to be the only important independent predictor, as it increases the probability of iSUA by 2.2 times according to the multivariable analysis (aOR: 2.234; 95% CI: 1.104–4.523) (Table 3).

The variance inflation factor was calculated among all the variables in every model; each value was close to 1, ruling out the possibility of multicollinearity. There were also no influential observations that could affect our results.

## 4. Discussion

### 4.1. Principal Findings

The main findings of this study were the following: (1) the incidence of prenatally diagnosed iSUA among singleton pregnancies is 1.3%, (2) there is a statistically significant association between iSUA and both SGA and PTD, suggesting that the prenatal diagnosis of this anatomical variation has clinical significance and should probably prompt closer surveillance in the third trimester, and (3) the only independent risk factor for the development of iSUA, constituting a novel finding, is conception via ART.

### 4.2. Interpretation of the Findings

Prenatally diagnosed iSUA affects approximately 1 in 100 singleton pregnancies, according to the findings of the present study; this is in accordance with the incidence calculated in a meta-analysis including 11 studies, which was equal to 1.2% [22]. Our study shows that singleton pregnancies with iSUA seem to have an almost two-fold increased risk for SGA neonates, compared to the singleton pregnancies with a three-vessel cord, when adjusted for all the possible confounders of maternal and obstetric characteristics. Our finding is consistent with the majority of published studies; the most recent meta-analysis of retrospective studies found that pregnancies with iSUA have a three-fold higher risk for SGA compared to singleton pregnancies with a three-vessel cord and may be associated with lower birthweight of about 200 g [22]. The authors of this meta-analysis highlighted the need for large prospective cohorts of high quality to further support this finding, perhaps due to the absence of a well-described pathophysiological mechanism explaining the association. Our study, although not large, is a prospective cohort of high quality and the detection of statistically significant associations in such a limited control group highlights the significant effect size between the two groups (aOR = 1.9). It is noteworthy that all the significant associations among the confounders and the risk of SGA are in accordance with the literature; low BMI, smoking, nulliparity and increased UtA PI have all been recognized as risk factors of SGA neonates [23,24,25]. In addition, a systematic review and meta-analysis by Luo et al. showed that, compared to normal neonates, those with iSUA had lower birthweight, worse Apgar score, increased risk of PTD, higher rate of cesarean section due to fetal distress, and increased rate of admission to neonatal intensive care unit (NICU), as well as prolonged NICU stay [11]. Similar results were found by another meta-analysis of a total of 1731 pregnancies with iSUA [26]. Moreover, a retrospective cohort study of 219 fetuses with iSUA and 219 controls using multivariable analysis that controlled for potential confounders also showed that iSUA is associated with SGA (aOR: 3.97; 95% CI: 1.55–10.12) [27]. Another retrospective cohort study, which compared 136 singleton pregnancies with iSUA with 500 consecutive singleton pregnancies with three-vessel cords, found that fetuses with iSUA are at higher risk of fetal growth restriction (15.4% vs. 1.8%; *p* < 0.001) and SGA (20.6% vs. 4.4%; *p* < 0.001) [28], and a case-control study concluded that this structural variation is also associated with SGA (14.3% vs. 4.9%; *p*  = 0.009) [29]. With regard to possible pathophysiology, it has been reported that reduced blood perfusion may not be the cause, as the SUA compensates with gradual dilation of the artery to decrease resistance and carry twice the blood volume [9]. A two-center prospective longitudinal observational study including 164 fetuses diagnosed with a SUA at the 20–22-week anomaly scan concluded that in most fetuses with iSUA, the remaining artery diameter at 20–22 weeks is significantly larger than in controls. However, when there are no changes in the diameter and in particular, if it remains <3.1 mm, the risk of abnormal fetal growth is higher [30]. Another hypothesis is that iSUA is associated with a greater risk of placenta abnormalities such as aberrant cord insertion; although we accounted for it without any important alteration in the result, abnormal cord insertion did not prove to be an important confounder in the multivariable models for either SGA or PTD [7,31]. An observational study of 34 cases with iSUA and 1799 cases with three-vessel cords, which underwent pathologic examination of the placenta after delivery, showed that the presence of iSUA in an SGA is associated with increased odds of high-grade fetal vascular malperfusion (aOR: 2.8; 95% CI: 1.1–7.5); this may partially explain the reason why iSUA carries a higher risk of SGA [32]. In addition, a meta-analysis proved that the presence of iSUA may increase the risk of perinatal complications such as SGA, oligohydramnios, polyhydramnios, gestational diabetes mellitus and perinatal mortality [33]. A study of 918,933 singleton pregnancies reported a 55% increased risk of SGA neonates in case of prenatally detected iSUA [7]. However, a thorough literature search revealed some controversial findings regarding the role of iSUA on perinatal outcomes. More specifically, a meta-analysis of three cohort and four case-control studies with a total of 928 pregnancies with iSUA failed to find a strong, statistically significant association between this structural variation and the risk for SGA [34]. Furthermore, another study concluded that serial antepartum ultrasound for fetal growth is not necessary in managing pregnancies complicated by iSUA, as this sonographic finding has a similar rate of SGA compared to a control group [9]. This statement was also endorsed by a prospective study of 138 cases with iSUA, which found that only 4 (2.9%) pregnancies were complicated by SGA [35].

Regarding PTD, the iSUA group had an almost doubled likelihood of having PTD compared to the controls. This finding is consistent with the existing literature; the most recent systematic review and meta-analysis showed that prenatally diagnosed iSUA carries a higher risk of PTD [22]. Several studies were found that investigated the association between PTD and prenatally diagnosed iSUA; Battarbee et al. adjusted for four confounders all included in our analysis and did not find a significant association [27], whereas the analysis by Hua et al. adjusted for three confounders and reached statistical significance [10]. Similarly, a retrospective study of 138 pregnancies with iSUA and 500 controls showed that fetuses with iSUA carry a higher risk of PTD (6.6% vs. 1.4%; *p* = 0.002), rendering this sonographic finding an independent risk factor for PTD (aOR: 5.0; 95% CI: 1.8–13.8; *p* = 0.002) [28]. Another study of 35,026 neonates with three-vessel umbilical cords and 223 neonates with SUA demonstrated that a pregnancy with iSUA is more likely to be complicated with PTD < 34 weeks (OR: 4.662; 95% CI: 2.346–9.195), polyhydramnios, SGA, cesarean section for fetal distress, perinatal death, admission to NICU and placental abnormalities, compared to a pregnancy with a three-vessel cord [36]. Possible important confounders that affected the association between PTD and iSUA, according to our model, were BMI, maternal age, smoking, conception via ART, history of PTD and UtA PI z-score. These associations have been previously confirmed by the currently existing literature [37,38]. Thus, a multivariable analysis to account for these confounders was implemented to allow the extraction of robust results.

As for preeclampsia, in the present study, no statistically significant association was identified between this pregnancy complication and iSUA. This finding is quite intriguing and in conflict with the most recently published meta-analysis on this topic, which found that iSUA is associated with an increased risk of pregnancy-induced hypertension (PIH) (OR: 2.23; 95% CI: 1.41–3.54; *p* = 0.0006) [22]. Another meta-analysis by Kim et al. supports the finding of the aforementioned study as it proved that iSUA increases the risk of PIH by about 60% (OR: 1.62; 95% CI: 1.00–2.63; *p* = 0.05) [26]. It is noteworthy that, in these meta-analyses, preeclampsia was not investigated as a distinct pregnancy outcome. On the contrary, a systematic review and meta-analysis of 15 studies, conducted by Xu et al., detected no strong association between iSUA and either preeclampsia or PIH, thus endorsing the finding of our study [33].

With regard to risk factors for iSUA, the only important individual risk factor was conception via ART. As maternal age becomes more advanced and subsequently the use of ART increases, the importance of this finding is clearly outlined. To date, no other study investigating the association between ART and iSUA has been found. Notably, a retrospective cohort investigated non-isolated SUA in singletons and ART and reported a statistically significant association [7]. The in vitro development of the chorion, which may influence placenta formation and predispose the woman to morphological abnormalities such as iSUA, is the most plausible explanation why ART could be a significant risk factor [39]. As for other potential risk factors, a meta-analysis found that maternal primigravidity and female gender of the neonate may be risk factors associated with iSUA [11], findings not confirmed by the present study.

To date, this is the first prospective cohort investigating the association of prenatally diagnosed iSUA with pregnancy outcomes, as well as possible risk factors. The greatest strength of this study lies in its design and methodology. The multivariable models that were carried out and reported according to the TRIPOD statement and the latest suggestions of the bibliography offer trustworthy and unbiased estimates. By further dealing with non-linearity and confirming the fulfilment of the regression assumptions, we took the extra step to ensure the robustness of our results. Additionally, the investigated pregnancy complications were meticulously selected based on their well-known correlation with adverse maternal and neonatal outcomes. More specifically, PTD is the main contributor to prolonged antenatal hospitalization, as well as the most significant cause of neonatal morbidity worldwide due to its association with intraventricular hemorrhage, retinopathy, necrotizing enterocolitis, acute respiratory distress syndrome and long-term neurologic impairment of the offspring, while it is also the second cause of infant mortality, following pneumonia [40]. With regard to SGA, these neonates carry an increased risk of both short- and long-term complications, such as perinatal asphyxia, impaired thermoregulation, hypoglycemia, feed intolerance, impaired immune function, polycythemia and neurodevelopmental impairment [41]. Including only prenatally diagnosed cases, this study may be considered useful to the maternal-fetal medicine specialists who are concerned about the implications of prenatal detection of iSUA and should follow an appropriate pregnancy surveillance protocol. Moreover, as previously mentioned, no large-scale studies evaluating the risk factors associated with iSUA exist, rendering our study a novelty. Our study’s primary weakness is its limited study group, considering that iSUA is an infrequent abnormality of the umbilical cord. Hence, unless further extensive prospective studies are carried out to allow for the extraction of safer and more reliable conclusions, our results should be interpreted with skepticism. Finally, even though only experienced maternal-fetal medicine specialists performed the scans, some cases of SUA could have been missed.

## 5. Conclusions

Isolated SUA is associated with an increased risk for SGA and PTD. This finding may justify an alteration in the routine obstetric management to include scanning of the umbilical cord vessels as a compulsory component and upon the diagnosis of iSUA a closer monitoring of fetal wellbeing in the third trimester may be warranted. ART, for the first time, is identified an independent predictor of the development of this anatomical variation. Further research into the detected associations and their pathophysiology is required.

## Figures and Tables

**Table 1:** Characteristics of the study population.

Characteristics	Overall (*n* = 6528)	Normal Cord (*n* = 6445)	iSUA (*n* = 83)	*p* Value
Maternal age, mean (SD)	31.7 (5.1)	31.7 (5.1)	32.4 (5.4)	0.205
No smoking, *n* (%)	4168 (63.8)	4112 (63.8)	56 (67.5)	0.358
Quit in pregnancy	1670 (25.6)	1654 (25.7)	16 (19.2)	
Current smoking	690 (10.6)	679 (10.5)	11 (13.3)	
Multiparous, *n* (%)	2620 (40.1)	2584 (40.1)	36 (43.4)	0.622
ART, *n* (%)	323 (4.9)	314 (4.9)	9 (10.8)	0.025 ^a^
BMI, median (IQR)	23.0 (21.0, 26.0)	23.0 (21.0, 26.0)	23.0 (21.1, 26.9)	0.767
Bleeding in 1st trimester, *n* (%)	331 (5.1)	327 (5.1)	4 (4.8)	1.000
Previous history of miscarriage in 1st trimester, *n* (%)	1030 (15.8)	1011 (15.7)	19 (22.9)	0.101
Previous history of PTD, *n* (%)	155 (2.4)	151 (2.3)	4 (4.8)	0.267
Abnormal cord insertion, *n* (%)	667 (10.2)	657 (10.2)	10 (12.0)	0.710
UtA PI z-score, mean (SD)	0.03 (1.10)	0.03 (1.10)	0.14 (1.07)	0.381
PE, *n* (%)	486 (7.4)	480 (7.4)	6 (7.2)	1.000
PTD, *n* (%)	558 (8.5)	544 (8.4)	14 (16.9)	0.011 ^a^
SGA, *n* (%)	1066 (16.3)	1043 (16.2)	23 (27.7)	0.008 ^a^
Gestational age at delivery, median (QR)	39.0 (38.1, 39.9)	39.0 (38.1, 39.9)	38.3 (37.6, 38.8)	<0.001 ^a^
BW, median (IQR)	3250 (2970, 3540)	3250 (2978, 3550)	3000 (2725, 3330)	<0.001 ^a^

Abbreviations: ART, assisted reproductive technology; BMI, body mass index; BW, birthweight; IQR, interquartile range; iSUA, isolated single umbilical artery; PE, preeclampsia; PTD, preterm delivery; SGA, small for gestational age; SD, standard deviation; UtA, uterine artery; PI: pulsatility index. ^a^ denotes statistically significant associations.

**Table 2:** Multivariable logistic regressions regarding the investigated outcomes.

	SGA		PTD	
Variables	aOR	95% CI	aOR	95% CI
Maternal age (years)	-	-	-	-
ΒΜΙ (kg/m^2^)	0.957	0.942, 0.972	1.021	1.003, 1.039
No smoking	reference		reference	
Quit smoking	-	-	-	-
Current smoking	1.844	1.507, 2.256	1.397	1.059, 1.842
Multiparity	0.634	0.544, 0.738	-	-
ART	-	-	2.033	1.436, 2.876
UtA PI z-score	1.560	1.470, 1.656	1.523	1.413, 1.642
Bleeding in 1st trimester	-	-	-	-
Previous history of miscarriage in 1st trimester	-	-	1.282	1.017, 1.616
Previous history of PTD	-	-	4.896	3.326, 7.209
Abnormal cord insertion	-	-	-	-
iSUA	1.909	1.152, 3.163	1.903	1.035, 3.498

Abbreviations: aOR, adjusted odds ratio; ART, assisted reproductive technology; BMI, body mass index; CI, confidence intervals; iSUA, isolated single umbilical artery; PTD, preterm delivery; SGA, small for gestational age; UtA, uterine artery; PI: pulsatility index. All the variables were included in the multivariable models; only statistically significant associations are presented.

**Table 3:** Risk factor analysis on the incidence of isolated single umbilical artery.

	Univariate Analysis			Multivariable Analysis		
Variables	OR	95% CI	*p*-Value	aOR	95% CI	*p*-Value
Maternal age (years)	1.028	0.985, 1.073	0.205	-	-	-
ΒΜΙ (kg/m^2^)	0.999	0.955, 1.046	0.981	-	-	-
No smoking	reference			reference		
Quit smoking	0.710	0.406, 1.242	0.230	-	-	-
Current smoking	1.190	0.620, 2.282	0.602	-	-	-
Multiparity	1.144	0.739, 1.772	0.545	-	-	-
ART	2.375	1.178, 4.788	0.016 ^a^	2.234	1.104, 4.523	0.025 ^a^
Bleeding in 1st trimester	0.947	0.345, 2.603	0.916	-	-	-
Previous history of 1st trimester miscarriage	1.596	0.952, 2.674	0.076	-	-	-
Previous history of PTD	2.110	0.763, 5.837	0.150	-	-	-
Male fetus	0.757	0.490, 1.170	0.210	-	-	-

Abbreviations: aOR, adjusted odds ratio; ART, assisted reproductive technology; BMI, body mass index; CI, confidence intervals; OR, odds ratio; PTD, preterm delivery. Only statistically significant associations are presented in the multivariable model. ^a^ denotes statistically significant associations.

## Data Availability

Data are available upon request.

## Supplementary Materials

The following supporting information can be downloaded at: https://www.mdpi.com/article/10.3390/medicina59061080/s1, Table S1: Univariate and multivariable analyses regarding small for gestational age neonates; Table S2: Univariate and multivariable analyses regarding spontaneous preterm delivery.
