# Supplementary material for: Prenatal Diagnosis of Isolated Single Umbilical Artery: Incidence, Risk Factors and Impact on Pregnancy Outcomes
Source: Medicina (Kaunas). 2023 Jun 3;59(6):1080. doi: 10.3390/medicina59061080 (PMC10305276; doi:10.3390/medicina59061080)
Supplement: Supplementary file 1 [file medicina-59-01080-s001.zip › medicina-2366266-supplementary.pdf]

Supplementary Table S1. Univariate and multivariable analyses regarding small for gestational age neonates

	**Univariate analysis**			**Multivariable analysis**		
Variables	OR	95% CI	p-value	aOR	95% CI	p-value
Maternal age (years)	0.997	0.984, 1.010	0.631	1.006	0.992, 1.020	0.409
BMI (kg/m^2)	0.959	0.945, 0.974	0.001[a]	0.957	0.942, 0.972	0.001[a]
No smoking		reference			reference	
Quit smoking	0.940	0.802, 1.101	0.442	0.961	0.816, 1.131	0.631
Current smoking	1.646	1.354, 1.999	0.001[a]	1.844	1.507, 2.256	0.001[a]
Multiparity	0.677	0.589, 0.778	0.001[a]	0.634	0.544, 0.738	0.001[a]
ART	1.103	0.823, 1.480	0.511	0.983	0.714, 1.355	0.918
UtA PI z-score	1.549	1.461, 1.643	0.001[a]	1.560	1.470, 1.656	0.001[a]
Bleeding in 1st trimester	0.953	0.703, 1.291	0.754	0.940	0.686, 1.289	0.701
Previous history of miscarriage in 1st trimester	1.093	0.917, 1.304	0.321	1.170	0.970, 1.411	0.101
Previous history of PTD	1.184	0.787, 1.783	0.418	1.531	0.994, 2.356	0.053
Abnormal cord insertion	1.316	1.074, 1.611	0.008[a]	1.180	0.955, 1.457	0.125
iSUA	1.958	1.222, 3.225	0.006[a]	1.909	1.152, 3.163	0.012[a]

Abbreviations: aOR, adjusted odds ratio; ART, assisted reproductive technology; BMI, body mass index; CI, confidence intervals; iSUA, isolated single umbilical artery; OR, odds ratio; PTD, preterm delivery; SGA, small for gestational age; UtA, uterine artery; PI: pulsatility index

[a] *denotes statistical significance*

Supplementary Table S2. Univariate and multivariable analyses regarding preterm delivery

	**Univariate analysis**			**Multivariable analysis**		
Variables	OR	95% CI	p-value	aOR	95% CI	p-value
Maternal age (years)	1.022	1.004, 1.039	0.014 [a]	1.003	0.984, 1.022	0.746
BMI (kg/m^2)	1.028	1.011, 1.045	0.001 [a]	1.021	1.003, 1.039	0.020 [a]
No smoking	reference				reference	
Quit smoking	1.165	0.954, 1.424	0.135	1.160	0.943, 1.427	0.160
Current smoking	1.367	1.046, 1.786	0.022[a]	1.397	1.059, 1.842	0.018[a]
Multiparity	1.085	0.910, 1.294	0.364	0.928	0.762, 1.131	0.458
ART	2.107	1.542, 2.880	0.001[a]	2.033	1.436, 2.876	0.001[a]
UtA PI z-score	1.527	1.417, 1.645	0.001[a]	1.523	1.413, 1.642	0.001[a]
Bleeding in 1st trimester	1.371	0.963, 1.952	0.080	1.293	0.896, 1.866	0.169
Previous history of miscarriage in 1st trimester	1.417	1.140, 1.761	0.002 [a]	1.282	1.017, 1.616	0.035[a]

Previous history of PTD	4.831	3.383, 6.898	0.001[a]	4.896	3.326, 7.209	0.001[a]
Abnormal cord insertion	1.460	1.131, 1.884	0.004[a]	1.232	0.944, 1.608	0.124
iSUA	2.201	1.231, 3.936	0.001[a]	1.903	1.035, 3.498	0.038[a]

Abbreviations: aOR, adjusted odds ratio; ART, assisted reproductive technology; BMI, body mass index; CI, confidence intervals; iSUA, isolated single umbilical artery; OR, odds ratio; PTD, preterm delivery; UtA, uterine artery; PI: pulsatility index

[a] *denotes statistical significance*
